# Neutrophil Maturity in Cancer

**DOI:** 10.3389/fimmu.2019.01912

**Published:** 2019-08-14

**Authors:** John B. G. Mackey, Seth B. Coffelt, Leo M. Carlin

**Affiliations:** ^1^Institute of Cancer Sciences, University of Glasgow, Glasgow, United Kingdom; ^2^Cancer Research UK Beatson Institute, Glasgow, United Kingdom

**Keywords:** neutrophil, cancer, myeloid, cancer inflammation, granulopoiesis

## Abstract

Neutrophils are implicated in almost every stage of oncogenesis and paradoxically display anti- and pro-tumor properties. Accumulating evidence indicates that neutrophils display diversity in their phenotype resulting from functional plasticity and/or changes to granulopoiesis. In cancer, neutrophils at a range of maturation stages can be identified in the blood and tissues (i.e., outside of their developmental niche). The functional capacity of neutrophils at different states of maturation is poorly understood resulting from challenges in their isolation, identification, and investigation. Thus, the impact of neutrophil maturity on cancer progression and therapy remains enigmatic. In this review, we discuss the identification, prevalence, and function of immature and mature neutrophils in cancer and the potential impact of this on tumor progression and cancer therapy.

## Introduction

Neutrophils in cancer have received very little attention until recently, despite contributing 50–70% and 10–25% of circulating leukocytes in humans and laboratory mice, respectively (1). However, recent progress has renewed interest in these cells. In experimental cancer models, neutrophils have been implicated in nearly every stage of the oncogenic process and their role has been reviewed in detail (2–4). Neutrophils are able to mediate a broad range of anti- and pro-tumor activities from direct cancer cell killing to tumor cell proliferation, angiogenesis, metastasis, and orchestrating other immune responses. These recent studies have highlighted the complexity of neutrophils in cancer progression, with novel information on their previously unappreciated plasticity and heterogeneity. While neutrophil plasticity can be directly affected by the local microenvironment, neutrophil heterogeneity is also influenced by their maturation (5), age (6), suppressive properties (7), function [e.g., phagocytosis (8)], and reverse transendothelial migration (rTEM) (9). An underexplored aspect of this is the appearance of immature neutrophils in cancer. Differences in the phenotype and functional capacities of immature and mature neutrophil populations are being identified, and their impact on cancer progression is emerging (10). However, the influence of neutrophil maturity on their anti- or pro-tumor properties remains understudied. In this review, we focus on the functional properties and relevance of immature neutrophils in cancer. We discuss methods used to identify neutrophils of different maturation states and explore their limitations. Finally, we postulate the impact that neutrophil maturity may have on the efficacy of cancer therapies.

## Granulopoiesis

After birth, neutrophil production occurs primarily in the bone marrow (BM) where they are derived from hematopoietic stem cells (HSCs). During neutrophil differentiation in mice and humans the nucleus progresses from a banded to segmented morphology, allowing the identification of neutrophils at distinct stages of maturity (11). Stages of neutrophil differentiation are also characterized by their unique expression of the transcription factors PU.1 and CCAAT enhancer binding protein (C/EBP)-α (12), C/EBPβ (13), and C/EBPε (14). Mature neutrophils are mitotically inactive with cell cycle arrest occurring during the myelocyte to metamyelocyte transition (15). The post-mitotic BM transit of neutrophils and release into the circulation takes between 5 and 8 days in humans (16) and 1–2 days in mice during homeostasis (17). Neutrophil granules, termed azurophillic (primary), specific (secondary), and gelatinase (tertiary), in addition to secretory vesicles, are formed at specific stages of neutrophil differentiation. Each granule type is composed of distinct proteins synthesized at the time of formation (18) and granules are released in reverse sequential order following neutrophil activation (19). As such, the proteome composition of immature and mature neutrophils is greatly different. It is important to also acknowledge that in disease, including cancer, granulopoiesis can occur outside of the medullary spaces of the BM, termed extramedullary hematopoiesis (EMH); however, little is known about the mechanisms regulating EMH and its influence on neutrophil development (20).

## Isolation and Identification of Immature Neutrophils

Despite the extensive data on neutrophils and their functions in homeostasis and disease, they remain a challenging cell population to study largely due to their short half-life [~18.5 h in the circulation of humans during homeostasis (16)] and propensity for priming and activation. While neutrophil life span can be increased following their activation and extravasation, a small window of opportunity for *in vitro* experimentation remains in comparison to other cell types. Neutrophil properties derived from *ex vivo* experimentation can be difficult to accurately interpret and apply to their behavior *in vivo*. Developments of *in vivo* imaging techniques and identification of neutrophils (e.g., via *in vivo* injection of fluorescently conjugated anti-Ly6G antibody, clone 1A8 (9, 21, 22) and fluorescent reporter mice (23) have allowed their investigation without possible *ex vivo* manipulation-induced artifacts; however, these approaches still have their own caveats for example the undetermined function for Ly6G (23–25). Importantly, experimental analysis of immature neutrophil populations is an even greater challenge.

### Density Properties

Neutrophil density changes during development as a result of their increased granularity and changes in cell size (26). Therefore, density gradient purification is useful for enriching neutrophil populations at certain stages of maturation and allows for down-stream analysis. Immature neutrophils are typically found in low density (LD) fractions, whereas mature neutrophils are found in the normal/high density (N/HD) fractions (5) (Tables 1, 2). Nevertheless, the neutrophil populations obtained by density gradient purification are not pure as N/HDNs can become LDNs following activation (55), making interpretation of the functional properties of neutrophil maturity challenging by this technique. For instance, LDNs isolated from the peripheral blood of 4T1 tumor-bearing mice make up ~40% of morphologically mature neutrophils (5), LDNs obtained from the peripheral blood of mice bearing breast cancer liver metastasis were composed of 80% neutrophils with an immature nuclear morphology (56), and the nuclear morphology of LDNs from lung cancer patients represent both mature and immature neutrophils (5). Overall, this technique can be useful for enriching neutrophil populations; although, more specific methods of identification of neutrophil maturity are required for accurate interpretation of downstream functional analysis.

**Table 1:** Methods for the identification of immature neutrophils in humans.

**Immature population**	**Feature/Cell surface markers**	**References**
Metamyelocyte Myelocyte	Sysmex IG	(27)
Myeloblast to mature	Low density	(28)
Immature	CD10^Low^CD15^High^	(29)
Myelocyte to band	Low density SSC^High^CD66b^Pos^CD125^Neg^ Pappenheim staining	(30)
MyeloblastPromyelocyte	Blood smears Celltac ES hematology analyser	(31)
Band	CD10^Dim^CD16^Dim^	(32)
Band	CD10^Dim^CD16^Dim^	(33)
Metamyelocyte Myelocyte Promyelocyte	CD11b^Low^CD16^Pos^	(34)
Immature	BM resident Nuclear Morphology	(35)
Metamyelocyte Myelocyte Promyelocyte	XE 2100, Sysmex hematology analyser	(36)
Band	CD16^Dim^	(37)
Metamyelocyte	CD35^Neg^CD49d^Pos^	(38)
Metamyelocyte Myelocyte Promyelocyte	Coulter Actdiff 5 automated hematology analyser	(39)
Immature	Nuclear morphology Number of nucleoli Cytoplasmic granularity	(40)

**Table 2:** Methods for the identification of immature neutrophils in mice.

**Immature population**	**Feature/Cell surface markers**	**References**
Myelocyte Meta-myelocyte	Nuclear morphology	(41)
Myeloblast	Nuclear morphology	(42)
Pro-myelocyte to band	Nuclear morphology	(43)
Band Meta-myelocyte	Nuclear Morphology Gr-1^Low^BrdU^Dim^	(17)
Mature Myelocyte Promyelocyte	Gr-1^Hi^CD11b^Pos^ Gr-1^Low^CD11b^Pos^	(44)
Band/mature	Nuclear morphology	(45)
Immature	Reduced MPO Reduced oxidative burst	(46)
Band	CD11b^Pos^Gr-1^Pos^Ly6G^Pos^Ly6C^Pos^MDL-1^Pos^	(47)
Band	Ly6G^Int^	(21)
Immature	Ly6G^Low/Neg^CD101^Neg^	(48)
Immature Myelocyte Pro-myelocyte	Gr-1^High^CD11b^Low^ Gr-1^Int^CD11b^Int^	(49)
Mature Band Myelocyte	Gr-1^Hi^ Gr-1^Low^	(50)
Neutrophil Precursors	Ly6G^Low^Ly6B^Int^CD115^Neg^ CD11b^Pos^CD133^Pos^	(51)
Mature Band	Gr-1^Hi^CD11b^Low−Hi^ Gr-1^Low−Hi^CD11b^Low−Hi^	(52)
Mature Band Metamyelocyte Myelocyte	Ly6G^Hi^CD11b^Pos^ Ly6G^Low^CD11b^Pos^	(53)
Mature Band Myeloblast Pro-myelocyte Myelocytes Meta-myelocyte	Lin^Neg^CD34^Low/Int^c- KIT/CD117^Neg^Ly6G^High^ Lin^Neg^CD34^Low/Int^c- KIT/CD117^High^Ly6G^Neg^ Lin^Neg^CD34^Low/Int^c- KIT/CD117^Int^Ly6G^Neg^ Lin^Neg^CD34^Low/Int^c- KIT/CD117^Int^Ly6G^Low^ Lin^Neg^CD34^Low/Int^c- KIT/CD117^Low^Ly6G^Int^	(13)
Metamyelocyte Myelocyte Promyelocyte Band Metamyelocyte Mature	Gr-1^Int^CD11b^Int^ Gr-1^Hi^CD11b^Low^ Gr-1^Hi^CD11b^Hi^	(54)

### Morphology and Cell Surface Markers

Nuclear segmentation is considered accurate for immature neutrophil identification in the peripheral blood of cancer patients (57) and mouse models of cancer (58) (Tables 1, 2). However, cells cannot be isolated by this method for downstream experimentation. A major hindrance in neutrophil biology is the lack of a specific and robust marker of neutrophil maturity. Changes in cell surface receptor expression during maturation, such as the CXCR4:CXCR2 axis (59, 60), can be used to separate immature and mature neutrophils (48) (Table 2). However, these surface receptors are prone to alteration following neutrophil activation [e.g., CD11b:CD18 (61)], tissue migration [e.g., CD62L (6, 62, 63)], and aging [e.g., CXCR4 (6)], resulting in a major challenge in the identification of efficient markers of maturity. In mice, immature and mature neutrophils can accurately be identified as Ly6G^Int/Low^CD11b^Pos^ and Ly6G^High^CD11b^Pos^ respectively (13, 21, 51) (Table 2). However, the limitations of using Ly6G as a maturity marker include relatively small differences in expression of this molecule between immature and mature neutrophils, compounding the technical issues associated with fluorescence intensity comparisons in some readouts. Despite this, recently identified markers of neutrophil maturity with larger differences in expression, for example CD101 (48), could be useful candidates for development of fluorescent reporter models and *in vivo* identification. Here, CD101 expression can be used to identify CD101^Neg^ (immature) and CD101^Pos^ (mature) neutrophils (48); however, this marker requires further validation to ensure its accuracy in a wide range of pathologies. Another example is c-KIT/CD117, the expression of which has been shown to associate with neutrophil maturity in naïve mice, mice undergoing candida-induced emergency granulopoiesis (13), and a mouse model of breast cancer (64) (Table 2). However, although in the *K14-Cre;Cdh1*^F/F^*;Trp53*^F/F^, and 4T1 mouse mammary tumor models, neutrophil c-KIT expression is enriched on immature neutrophils, it fails to completely correlate with maturation status (58, 65). In humans, immature and mature neutrophils are commonly identified as CD16^Low^CD10^Neg^ and CD16^High^CD10^Pos^, respectively (66) (Table 1). Expression of CD16 (FcγRIII) is initiated between the metamyelocyte and band stages of neutrophil maturation (67, 68). However, its expression can be reduced during apoptosis (69) and can be up-regulated on the cell surface following secretory granule cell membrane fusion (67). Distinct differences in the hematopoietic environment, local and systemic cytokine levels and the functional requirements for neutrophils will exist between naïve, emergency granulopoiesis and the more chronic “inflammation” present in cancer. Therefore, as neutrophils can exhibit plasticity in response to their environment, certain markers are likely to only be suitable in particular models and require efficient validation in each. Overall, the challenges associated with identifying and isolating populations of neutrophil maturity have hindered their study and our current understanding of their functional properties.

## Functional Properties of Mature and Immature Neutrophils

### Immature Neutrophils in Cancer

The existence of immature neutrophils in the circulation and tissues is a consequence of cancer development in human patients and mouse models. For example, immature neutrophils are detectable in the circulation (and in some cases the primary tumors) of both injectable and transgenic mouse models of colon (70), skin (70), mammary (5, 58, 71, 72), lung cancer (5, 73), and mesothelioma (AB12) (5, 73). In humans, immature neutrophils have been described in patients with lung cancer (5, 74), breast cancer (5), and ovarian cancer (65).

### Drivers of Immature Neutrophil Appearance Outside of the Hematopoietic Niche

**Figure 1:**
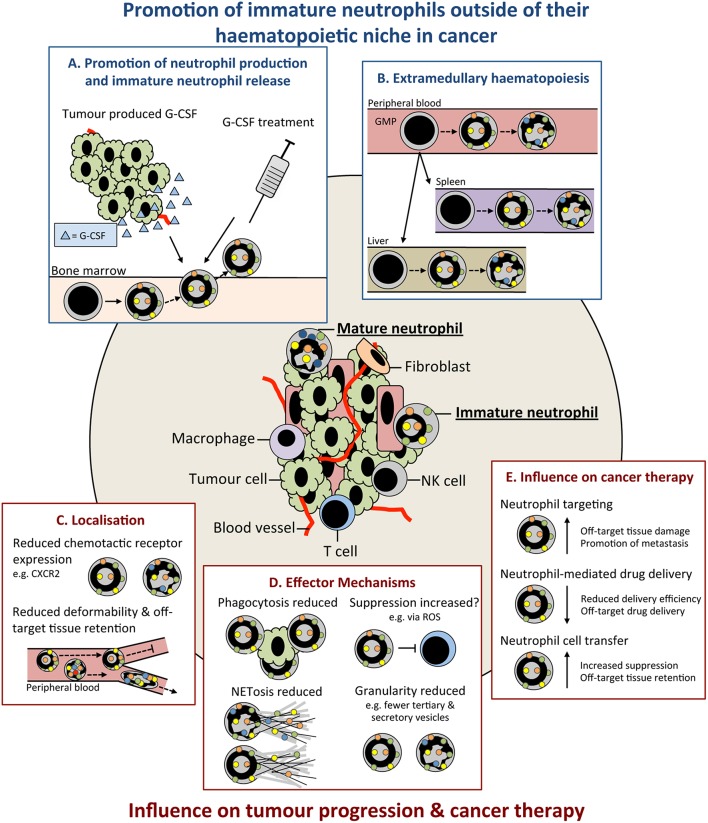
Immature neutrophils are present in cancer and have an altered functional capacity compared to mature that may influence tumor progression. Immature neutrophils can be present and significantly increased in the peripheral blood and tissues of cancer patients. This increase may result from: **(A)** promotion of their early release from their bone marrow (BM) haematopoietic niche by increased systemic chemokines, such as granulocyte colony-stimulating (G-CSF), e.g., tumor produced or as therapy. **(B)** release of neutrophil precursors from the BM and their extramedullary proliferation in the circulation or tissues. Immature neutrophils may have both anti- and pro-tumor properties. These include **(C)** altered localization resulting from their differential cell surface marker expression influencing their chemotactic capacity and/or less segmented nuclear morphology compared to mature neutrophils reducing their deformability and **(D)** different functional capacity compared to mature neutrophils including their reduced phagocytic capacity, altered suppressive properties, reduced NETosis, and reduced granularity. **(E)** Together, these differences in the properties and functions of immature neutrophils could lead to their negative influence when targeting neutrophils in cancer therapy. G-CSF, colony stimulating factor-3; GMP, granulocyte monocyte progenitor; ROS, reactive oxygen species; NET, neutrophil extracellular trap; NK, natural killer cell.

Premature release from the BM as observed in states of emergency granulopoiesis, as reviewed by others (75), is considered the main reason for the presence of immature neutrophils in the circulation. Emergency granulopoiesis commonly results from increased levels of granulocyte colony-stimulating factor (G-CSF; also known as CSF-3) (76) that promotes the differentiation of hematopoietic precursors down the neutrophil lineage and release of neutrophils into the circulation (60, 71, 72, 77) (Figure 1A). Production of G-CSF is controlled by interleukin (IL)-23 and IL-17 (58, 78, 79) and can be increased in many cancer models and patients (56, 58, 71, 72, 80). Enhanced levels of G-CSF drive excessive production and release of neutrophils and their precursors into the circulation, leading to neutrophilia (58, 71, 72, 81). G-CSF is dispensable for emergency granulopoiesis and other cytokines, including granulocyte/macrophage (GM)-CSF (also known as CSF-2) (43, 75), can drive neutrophil production and release. Furthermore, neutrophil precursors can seed distant tissues and produce neutrophils *in situ*, as has been observed in cancer patients (82) (Figure 1B). TGFβ is another cytokine that favors the presence of immature neutrophils, since its inhibition converts neutrophils to a mature phenotype in transplantable models of mesothelioma (73). The N1/N2 nomenclature—which mirrors the Th1/Th2 nomenclature of T helper cells—was coined in this study based on the influence of TGFβ to modulate neutrophil phenotype and function. Neutrophils were named pro-tumor N2 cells or anti-tumor N1 cells after Th1/Th2 CD4 T cells and M1/M2 macrophages. However, evidence that neutrophils mediate type 1 or type 2 immunity is lacking, and additionally, how these phenotypes relate to the neutrophils found in patients is still under investigation [recently reviewed in Shaul and Fridlender (83)]. Therefore, this nomenclature may be confusing in the context of cancer at this time and future work will determine its appropriateness. In contrast to TGFβ, expression of Type 1 interferons (IFNα and IFNβ) in tumors favors mature neutrophils over immature neutrophils (57). Most likely, there are many other tumor-derived factors that influence neutrophil maturity and their discovery could lead to opportunities for therapeutic intervention.

### Functional Properties of Immature Neutrophils

The degree of functional difference between immature and mature neutrophils remains an open question in the field. Due to the importance of neutrophil differentiation in their effector mechanisms, (e.g., production of granule proteins), there is a strong argument for functional differences. Immature neutrophils may in many cases fall under the myeloid-derived suppressor cell (MDSC) umbrella, as these cells have been reported to inhibit T cells. MDSCs encompass a wide range of granulocytic and monocytic cell types at different stages of differentiation. Polymorphonuclear (PMN)-MDSCs are widely considered to be an immature neutrophil population, but methods for their identification, including with anti-Gr-1 (clone RB6-8C5) antibody—which recognizes both Ly6C and Ly6G epitopes—fail to accurately discriminate between mature and immature cells (84, 85). Recently, the classification and identification of MDSC subsets based on their phenotype and morphology has been improved, but these are still identified as CD11b^Pos^Ly6C^Low^Ly6G^Pos^ (84). Nevertheless, we believe that the suppressive functions of immature and mature neutrophils is a pathological response to tumorigenesis rather than a completely separate granulocytic population, as discussed by others (86–88). Therefore, we refer to PMN-MDSCs as neutrophils in this article. *Ex vivo* suppression assays are the most common technique for identifying and analyzing suppressive neutrophils. Findings that have used this technique are challenging to interpret as they can be influenced by neutrophil survival, cytotoxicity, neutrophil:T cell ratio, and protocols used [e.g., CD3/CD28 microbeads or antibodies (89)]. Immature (90) and mature (91) neutrophils can be suppressive; however, differences in the suppressive capacities of these populations (66, 92) are likely influenced by disease, model, and neutrophil isolation and identification protocols used (Figure 1D). It should also be noted that not all tumor-infiltrating immature neutrophils possess T cell-suppressive abilities (93, 94).

Immunosuppression by neutrophils is not only important for primary tumor progression, but this mechanism can also promote metastasis formation. Neutrophils can be recruited by CXCR2 ligands to dampen anti-tumor immunity in pre-metastatic organs so that disseminated cancer cells can evade immune destruction (58, 95, 96). In these cases, it is the immature neutrophils that are thought to mediate immunosuppression and subsequent metastasis; although, this has not been formally shown. In addition, immature neutrophils and other myeloid progenitors can aid in the formation of the pre-metastatic niche via mechanisms other than T cell suppression (97–99). Interestingly, in models where immature neutrophils are absent, such as the *MMTV-PyMT* model of breast cancer, it is the mature neutrophils that drive metastasis (100). Together, these data indicate that neutrophil maturity may be irrelevant to their pro-metastatic functions.

ROS production is important in several neutrophil effector mechanisms including their microbicidal (101), phagocytic (102) and suppressive capacity and contributes to neutrophil anti- and pro-tumor functions [reviewed in Ohl and Tenbrock (103)]. One such pro-tumor function of neutrophil ROS in cancer is their promotion of tumor initiation at states of inflammation by damaging proliferating epithelial cells (104). In relation to neutrophil maturity the production of ROS can be variable between immature and mature cells. For example, immature neutrophils (Ly6G^Pos^CD101^Neg^) display reduced ROS production compared to mature (Ly6G^Pos^CD101^Pos^) in a mouse orthotopic pancreatic cancer model (48). Similarly, in a range of other transplantable mouse cancer models, LDNs—which are enriched in morphologically immature neutrophils—have reduced ROS production (5). However, the amount of ROS production may be context dependent and reliant on metabolism. In tumor-free mice (Ly6G^Int^c-Kit^Pos^) and ovarian cancer patients (CD10^Int^), immature neutrophils are dependent on oxidative mitochondrial metabolism rather than glycolysis, for ROS production (65). Recently, LDNs from mice bearing 4T1 mammary tumor liver metastasis have also been shown to have an increased oxidative metabolism (56). This dependency may have implications in the glucose-limited tumor microenvironment and affect neutrophil function (65). Therefore, while immature neutrophils can have reduced ROS production compared to mature neutrophils, this appears to be dependent on stimulus, their localization and the tissue microenvironment.

Neutrophil extracellular traps (NETs) are extracellular fibers composed of nuclear, mitochondrial, cytoplasmic and granule contents that can be released by neutrophils following their activation (105). NETs can capture circulating cancer cells in the mouse lung promoting their extravasation and metastasis formation (106, 107). Neutrophils can also aid in formation of the omental pre-metastatic niche and capture of circulating ovarian cancer cells, promoting their metastasis at this site (108). The ability of immature human neutrophil populations to release NETs is reduced following interferon priming (35) (Figure 1D). In addition, when isolated from the peripheral blood of acute myeloid leukemia (AML) patients, morphologically immature neutrophils show decrease capacity for NET formation following phorbol 12-myristate 13-acetate (PMA) stimulation (109). As NETs have been proposed to arise from the inability of terminally differentiated neutrophils to re-enter mitosis (110), it could be inferred that the increased mitotic capacity of immature populations contributes to these differences. ROS contribute to NETosis by promoting granule release and rupture of the nuclear envelope, as highlighted by the inability of neutrophils from chronic granulomatous disease patients to undergo NETosis (111, 112). Differences in ROS production with neutrophil maturity may also influence NETosis (65). Differences in granule composition of neutrophils at different maturity may also influence the functional capacity of their NETs. The tertiary granule component MMP-9 (113) has been implicated in NET-induced dormant cancer cell reactivation (114) and its possible reduced abundance in banded neutrophils and earlier neutrophil precursors present in cancer could reduce their ability to promote this reactivation.

Multiple studies have indicated a reduced migratory capacity of immature compared to mature neutrophils (5, 48) (Figure 1C). This may result from lower expression of chemokine receptors, such as CXCR1 and CXCR2 (30), and other genes involved in chemotaxis (48). In mice, proliferating neutrophil precursors, identified as Ly6G^Low^CXCR2^Neg^c-Kit^Pos^CXCR4^Pos^, have reduced migration to laser-induced damage (48). High CXCR2 expression by neutrophils has been associated with poor outcome in human pancreatic ductal adenocarcinoma (PDAC) patients (95). Inhibition of CXCR2 in a mouse model of PDAC reduces neutrophil migration and delays tumor progression (95). Banded nuclear morphology, and thus reduced deformability, may promote immature neutrophil sequestration in capillaries and reduce their migratory capacity (115); although, banded nuclear morphology in immature human neutrophils does not affect transendothelial migration (TEM) when compared to segmented neutrophils *ex vivo* (62) (Figure 1C). It is therefore conceivable that their increased sequestration in off target tissues and ability to undergo TEM may result in unwanted immature neutrophil accumulation and the promotion of inflammation and/or metastasis. Additionally, neutrophil spontaneous migration is increased in the early compared to late stages of cancer in a mouse orthotopic lung cancer model (116). These changes in neutrophil function with tumor progression are present in BM cells, suggesting altered granulopoiesis over time (116). Therefore, while further investigation is required, differential trafficking of immature neutrophils could have the capacity to both antagonize and promote tumor development dependent on their localization.

The phagocytic capacity of immature, compared to mature, neutrophils is also reduced (5, 48) and could result from their altered cell surface receptor expression and decreased ROS production (Figure 1D). Fc receptors (FcRs) are important in mediating phagocytosis (117) with decreased expression of CD16 likely influencing their phagocytic capacity. Furthermore, immature neutrophils (CD16^Int^) are unable to kill tumor cells via FcγRI, but exhibit cytotoxicity via FcαRI (118). Activation of FcRs, integrins and G-protein coupled receptors (GPCRs) can trigger neutrophil ROS production and its extracellular or intracellular release into the phagolysosome, as reviewed in more detail by others (102, 119). Unsurprisingly, immature neutrophils have also been shown to have an increased life span and can mature *ex vivo* (120). It will be interesting to determine if neutrophil maturation after their release from the BM contributes to heterogeneity within the mature neutrophil population. However, despite differences in the functional capacity of immature and mature neutrophils, they are still capable of mediating innate immune functions (120). Overall, the effect of neutrophil maturity in cancer remains enigmatic and further investigation, coupled with accurate identification, is required.

## Neutrophil Maturity in Anti-Cancer Therapy

Immunotherapy has shown great promise in cancer; however, only a minority of patients respond to certain therapies (121) and combinatorial therapies targeting a broad range of immune populations may be more beneficial. Therapies targeting neutrophils have received relatively little attention (Figure 1E). While the direct effect of therapies on neutrophils at different stages of maturation has not been investigated, we can consider ways in which the properties of immature neutrophils are relevant.

Neutrophils recruited to the tumor via CXCR2 can aid tumor progression (122) and inhibition of CXCR1 and CXCR2 has shown promise in mouse models (95) and human cancers (123). As CXCR2 expression increases with neutrophil maturation (48) inhibitors of CXCR2 may differentially influence immature and mature neutrophils affecting their efficacy. Therapies targeting immunosuppressive neutrophils enhance responses to checkpoint blockade by promoting tumor infiltration by T cells in mouse models (124–127). A greater understanding of the maturity composition of these cells could better aid targeting of this population. Furthermore, tyrosine kinase inhibitors that target the hepatocyte growth factor (HGF) receptor, cMET (e.g., Cabozantinib and Capmatinib) can extend survival by influencing neutrophil behavior in mouse melanoma and PTEN/p53-deficient prostate cancer models (128, 129). As tyrosine kinases (e.g., Bruton's tyrosine kinase; BTK) are important in regulating neutrophil development (49) and in neutrophil integrin signaling (130) it is possible that their inhibitors have altered effects on neutrophils of different maturity. Similarly, monoclonal antibody (mAb) based therapies, for example anti-gp75 (TA99) (131), anti-HER2 (Trastuzumab) (131), and anti-SIRPα (KWAR23) (132, 133), promote neutrophil-mediated destruction of cancer cells. The reduced phagocytic capacity (48) of immature neutrophils and differences in their FcR expression (118) may reduce the efficacy of these therapies. Furthermore, these properties may hamper their ability to deliver therapeutics, such as in nanoparticles (134), to the tumor (Figure 1E). Finally, adoptively transferred neutrophils can aid in the killing of cancer cells (135) and can be isolated from G-CSF-treated donors (136) (Figure 1E). Here, the activation (137) and potential retention of transferred immature neutrophils in off-target organs [e.g., the lung (138)] needs to be considered. In addition, G-CSF-driven immature neutrophil release, neutrophil accumulation, and alterations to neutrophil function in cancer (66, 100, 139) need to be further deliberated when treating neutropenic cancer patients with G-CSF (140, 141).

## Conclusions

To gain an accurate understanding of maturity on neutrophil functional capacity, consensus protocols for identification of neutrophil maturity are urgently required. However, as protocols and markers may not be transferable between models, detailed confirmation of maturity in each is essential (e.g., associated nuclear morphology, transcriptomics, proteomics, and surface protein expression data) allowing for proper comparison. Functional investigation needs to be further driven by *in vivo* investigation to remove concerns associated with *ex vivo* manipulation. Of particular importance, investigating the localization and suppressive capacity of immature neutrophils *in situ* will aid in determining their influence on immunotherapy. Furthermore, more research on immature neutrophils in cancer patients should be carried out to determine where these cells appear. Correlations between immature neutrophils and mutational drivers need investigation to understand how these cells occur outside the bone marrow and to identify additional biomarkers of disease. Changes in neutrophil maturation status before, during and after anti-cancer therapy may provide insight into how these cells are regulated. Taken together, the available evidence suggests immature neutrophils in cancer inevitably influence tumor development and we emphasize the importance of improving methodologies for their study.

## Author Contributions

JM, SC, and LC wrote and edited the manuscript.

### Conflict of Interest Statement

The authors declare that the research was conducted in the absence of any commercial or financial relationships that could be construed as a potential conflict of interest.
